# Deepening the understanding of the structural validity of the Tilburg Frailty Indicator

**DOI:** 10.1007/s40520-023-02407-w

**Published:** 2023-04-21

**Authors:** Mercè Balasch-Bernat, Trinidad Sentandreu-Mañó, José M. Tomás, Maria A. Cebrià i Iranzo, Maria A. Tortosa-Chuliá, Anna Arnal-Gómez, Natalia Cezón-Serrano

**Affiliations:** 1 Department of Physiotherapy, University of Valencia, C/Gascó Oliag, 5, 46010 Valencia, Spain; 2 Physiotherapy in Motion. Multi-Speciality Research Group (PTinMOTION), Department of Physiotherapy, University of Valencia, 46010 Valencia, Spain; 3 Advanced Research Methods Applied to Quality of Life Promotion (ARMAQoL), University of Valencia, 46010 Valencia, Spain; 4 Department of Methodology for the Behavioral Sciences, University of Valencia, 46010 Valencia, Spain; 5 Physical Medicine and Rehabilitation Service, La Fe Hospital in Valencia, La Fe Health Research Institute (IISLAFE), 46026 Valencia, Spain; 6 Department of Applied Economics, University of Valencia, 46022 Valencia, Spain; 7 Public Economic Evaluation Research Group (EVALPUB), Department of Applied Economics, Faculty of Economics, University of Valencia, 46022 Valencia, Spain

**Keywords:** Frailty, Older adults, Psychometrics, Structural validity

## Abstract

**Background:**

Psychometric properties of the Tilburg Frailty Indicator (TFI) have shown low internal consistency for psychological and social domains, and evidence for its structure validity is controversial. Moreover, research on TFI is frequently limited to community dwellings.

**Aims:**

To evaluate structural validity, reliability, and convergent and divergent validity of the Spanish version of the Tilburg Frailty Indicator (TFI) in both community-dwelling and institutionalized older people.

**Materials and methods:**

A cross-sectional study was conducted on Spanish older adults (*n* = 457) recruited from both community settings (*n* = 322) and nursing homes (*n* = 135). Participants completed the TFI and other frailty instruments: Fried’s Frailty Phenotype, Edmonton Frailty Scale, FRAIL Scale, and Kihon Checklist (KCL). Confirmatory Factor Analysis (CFA), and reliability and validity coefficients were estimated.

**Results and discussion:**

Some items from physical and social domains showed low factor loadings (< 0.40). The three-factor CFA model showed better fit indices after depurating these items. Reliability estimates were good (CRI ≥ 0.70) for physical and psychological domains in the institutionalized sample, while in the community dwellings, only physical domain reliability was adequate. Convergent and divergent validity of physical and psychological domains was good, except for some alternative psychological measures highly correlating with the TFI physical component (KCL-depressive mood and Edmonton mood). However, the social domain showed low correlations with some social indicators.

**Conclusion:**

The findings of this study clarify some of the controversial validation results of the TFI structure and provide evidence to improve its use in psychometric terms.

**Clinical trial registration number:**

NCT03832608.

## Introduction

Studies on frailty are increasing in literature on aging. There is no consensus on its definition, but it is generally recognized as a state of increased vulnerability that is associated with high risk of adverse outcomes, such as falls, disability, and even mortality [1, 2]. Traditionally, frailty was considered a unidimensional physical construct [3]. A broader paradigm is supported by other researchers who refer to a multidimensional approach with physical, psychological, and social factors, which interact and disturb the physiological balance [4].

Within this construct, the Tilburg Frailty Indicator (TFI) is a multi-domain frailty instrument, developed in 2010 as a screening tool for frailty [4, 5]. It has been translated and validated into multiple languages as Portuguese [6, 7], Polish [8, 9], Italian [10], German [11], Danish [12], Spanish [13], Arabic [14], Persian [15], Greek, and Croatian [16]. Several authors have reported low internal consistency estimates for the psychological and particularly the social frailty domains [4, 6, 7, 9–11, 13, 14, 16–18]. Additionally, lower predictive capacity has been found for the psychological and social components, especially the social one [19, 20].

Among all validations, only the Spanish [13], Turkish [18], and Taiwanese [21] studies have analyzed TFI’s structural validity. Confirmatory Factor Analysis (CFA) gave some support for the three domains of frailty but found poor indicators of the physical and the social domain with low factor loadings [13, 18, 21], suggesting that the TFI model in its current form is not entirely supported by the data [13]. In addition, the Turkish CFA also had some limitations, as no information about the estimation method or the correlations among factors was provided. On the other hand, a recent systematic psychometric review [22] of this measurement instrument concludes that, despite the large number of validation studies available, it is necessary to continue accumulating evidence on metric properties such as the structural validity of this tool.

Additionally, research on TFI has been limited to community-dwelling older adults. Thus, further studies involving institutionalized older adults could contribute to test its applicability to other contexts [15, 22]. Therefore, the aim of this study is to further validate the Spanish version of the TFI by Vrotsou et al*.* [13] in both institutionalized and community-dwelling older adults. The following psychometric properties will be assessed, with an emphasis on the factor structure including: (1) structural validity; (2) internal consistency; and (3) convergent and divergent validity.

## Materials and methods

### Population and study design

This cross-sectional study was carried out between 2018 and 2021. A convenience sample of 457 older adults aged ≥ 65 years was included. Community-dwelling older adults were recruited from several community settings (*n* = 322), and institutionalized participants from nursing homes (*n* = 135). Exclusion criteria included Mini-Mental State Examination < 18 points, acute disease, inability to walk, and hospital admission or unstable chronic disease in the last month. All participants signed an informed consent form. Ethical approval was given by the Ethics Committee for Human Research of the University of Valencia (H1542733812827). The research was conducted in accordance with the principles of the Declaration of Helsinki and was registered at http://www.clinicaltrials.gov (ID: NCT03832608).

### Measurements

Tilburg Frailty Indicator was measured with a 15-item questionnaire, addressing physical (8 questions), psychological (4 questions), and social domains (3 questions). All items were dichotomized and scored with 0 points (absence) or 1 point (presence), and summed to obtain the total score ranging from 0 to 15 [4].

Alternative frailty assessment tools were included: Fried’s Frailty Phenotype [3] has five criteria assessing unintentional weight loss, exhaustion, low physical activity, reduced grip strength, and reduced gait speed; the Edmonton Frailty Scale [23] evaluates nine domains of frailty: cognition, general health status, functional independence, social support, medication usage, nutrition, mood, continence and functional performance; the FRAIL Scale [24] is a five-item screening tool including fatigue, resistance, ambulation, illness, and weight loss components; and finally, the Kihon Checklist (KCL) [25] is a self-report multidimensional screening tool with seven domains: instrumental activities of daily living, physical strength, nutrition, eating, socialization/isolation, memory, and mood. All participants were interviewed for the questionnaire’s completion and assessed for physical tests by trained researchers in a single session.

### Statistical analysis

SPSS 26 was used to calculate descriptive statistics for the variables under study, and to obtain Cronbach’s alpha coefficients, corrected item-total correlations, and correlations among the dimensions in the TFI and external criteria. Additionally, an R function was used for alpha coefficients confidence intervals. Given the nature of the study with voluntary participation and interviewers present, there was a very low percentage of missing data. There was only one missing data point (0.2%), from the institutionalized sample in a single indicator. With such very low level of missingness in the datasets, there is no need to handle the missing data, and therefore list-wise selection was employed across the statistical analyses. The factor structure was tested with CFAs estimated with Weight Least Square Mean and Variance (WLSMV) corrected estimation in Mplus 8.6. WLSMV was selected because the variables are binary and lacked multivariate normality. Several fit indices were used for assessing model fit: Chi-square statistic; Comparative Fit Index (CFI); Root Mean Square Error of Approximation (RMSEA); and Standardized Root Means-square Residuals (SRMR). Criteria for reasonable fit were [26]: a CFI of at least 0.90, and a RMSEA and SRMR less than 0.08 together, indicate adequate fit. The Composite Reliability Index (CRI) for each dimension in the scale was calculated, as a superior measure of internal consistency compared to alpha. Values ≥ 0.70 represent good internal consistency [27].

Finally, Spearman’s correlations were used to study the convergent and divergent validity of the physical, psychological, and social domains of the TFI with other frailty assessment tools. Based on Cohen’s criteria, a correlation coefficient of 0.10 ≤ 0.30, 0.30 ≤ 0.50, and ≥ 0.50 indicated weak, moderate, and strong correlations, respectively [28]. Additionally, very similar guidelines are those in the COSMIN guide: adequate validity is shown if *r* ≥ 0.50 for similar constructs, *r* = 0.30–0.50 for related constructs, and *r* < 0.30 for unrelated constructs.

## Results

Descriptive statistics are presented as means and standard deviations or percentages for the variables in Table 1, and for each of the items of the TFI in Table 2.

**Table 1:** Main descriptive characteristics of the sample (*n* = 457)

Characteristic	Mean ± SD, median (range) or *n* (%)	
	Community-dwelling older adults (*n* = 322)	Institutionalized older adults (*n* = 135)
Age (years)	72.5 ± 5.7	81.9 ± 8.4
Gender (women)	222 (68.9)	103 (76.3)
Marital status (married)	193 (59.9)	18 (13.3)
Level of education		
None/primary	98 (30.5)	107 (79.3)
Secondary	12 (3.7)	9 (6.7)
Higher	211 (65.5)	19 (14.1)
No. prescribed medications	3.5 ± 2.6, 3 (0–15)	8.7 ± 4.3, 8 (0–26)
No. falls in the last year	0.5 ± 0.9, 0 (0–7)	1.1 ± 2.1, 1 (0–14)
No. hospital admissions in past year	0.1 ± 0.4, 0 (0–2)	0.3 ± 0.6, 0 (0–4)
Comorbidity		
Musculoskeletal	267 (82.9)	83 (61.5)
Respiratory	41 (12.7)	28 (20.7)
Cardiovascular	149 (46.3)	101 (74.8)
Endocrine-metabolic	157 (48.8)	85 (63)
Neurological	48 (14.9)	64 (47.4)
Gastrointestinal	99 (30.7)	46 (34.1)
Renal	43 (13.4)	43 (31.9)
Others	141 (43.8)	74 (54.8)
Economic status		
I live well	250 (77.6)	24 (17.8)
I can deal with basic needs	68 (21.1)	95 (70.4)
I have difficulty dealing with basic needs	4 (1.2)	13 (9.6)
I cannot deal with basic needs	0 (0)	3 (2.2)
Barthel Index (0–100)	97.9 (3.7)	77.9 (19.0)
Tilburg Frailty Indicator (0–15)	4.3 ± 2.6	5.5 ± 3.5
Physical domain	2.2 ± 1.7	3.2 ± 2.3
Psychological domain	1.3 ± 1.0	1.8 ± 1.3
Social domain	0.8 ± 0.8	0.5 ± 0.7
Fried’s Frailty Phenotype (0–5)	0.8 ± 0.9	1.9 ± 1.1
Edmonton Frailty Scale (0–17)	2.7 ± 2.1	7.6 ± 2.4
FRAIL Scale (0–5)	0.6 ± 0.8	2.0 ± 1.2
Kihon Checklist (0–25)	4.6 ± 3.3	12.2 ± 3.7

**Table 2:** Descriptive data for the TFI items

TFI items	*n* (%)	
	Community-dwelling older adults (*n* = 322)	Institutionalized older adults (*n* = 135)
Item 1. Feeling physically healthy (no)	38 (11.8)	37 (27.4)
Item 2. Involuntary loss of weight (yes)	11 (3.4)	34 (25.2)
Item 3. Difficulty in walking (yes)	78 (24.2)	68 (50.4)
Item 4. Difficulty maintaining your balance (yes)	99 (30.7)	61 (45.2)
Item 5. Poor hearing (yes)	107 (33.2)	52 (38.5)
Item 6. Poor vision (yes)	148 (46.0)	68 (50.4)
Item 7. Lack of strength in your hands (yes)	105 (32.6)	55 (40.7)
Item 8. Physical tiredness (yes)	106 (32.9)	52 (38.5)
Item 9. Memory problems (yes)	71 (22.0)	55 (40.7)
Item 10. Feeling down (yes)	148 (46.0)	81 (60.0)
Item 11. Feeling nervous or anxious (yes)	157 (48.8)	70 (52.2)
Item 12. Being able to cope with problems (no)	29 (9.0)	37 (27.4)
Item 13. Living alone (yes)	101 (31.4)	8 (5.9)
Item 14. Missing having people around (yes)	142 (44.1)	41 (30.4)
Item 15. Receiving enough support (no)	27 (8.4)	19 (14.1)

### Structural validity

Two CFA models were estimated in both community-dwelling and institutionalized older adults. These models were: a one-factor solution (frailty); and a three-factor solution with the three frailty domains: physical (items 1–8), psychological (items 9–12), and social (items 13–15).

**Fig. 1:**
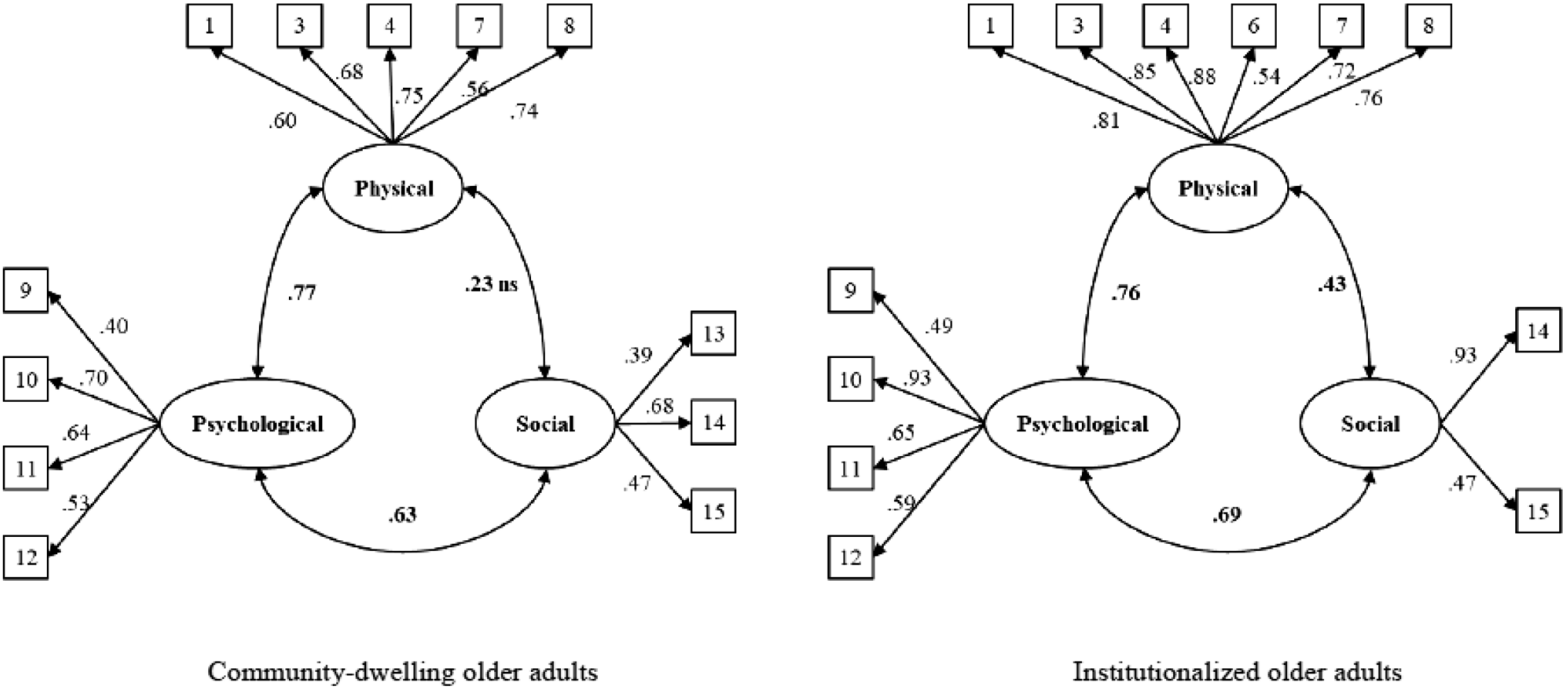
Final confirmatory factor analysis (CFA) standardized parameter estimates for the Tilburg Frailty Indicator in community-dwelling and institutionalized older adults

The one-factor solution had a poor fit: *χ*^2^(90) = 167.60, *p* < 0.001; RMSEA = 0.052; CFI = 0.847; and SRMR = 0.102. The three-factor model had better fit, but was still unsatisfactory: *χ*^2^(87) = 138.06, *p* < 0.001; RMSEA = 0.043; CFI = 0.899; and SRMR = 0.099. Additionally, no theoretically sound modification index could help in terms of fit. The factor loadings for items 2, 5, and 6 in the physical domain were all lower than 0.4. When deciding for this limit, it must be borne in mind that a factor loading of 0.4 indicates that only a 16% of the variance of the indicator is shared with the dimension that pretends to measure. Specifically, the standardized factor loadings for the three items were: 0.057 (*p* = 0.763) for item 2; 0.186 (*p* = 0.028) for item 5; and 0.372 (*p* < 0.001) for item 6. Apart from the statistical considerations of low relation with the dimension, there are substantive reasons that may also explain why these items behaved poorly. Regarding item 2, maybe it is difficult for an old adult to estimate what is an involuntary large amount of weight loss. Regarding items 5 and 6, they recall worsening of audition and vision, respectively. A worsening of these conditions is natural in the old age, but it may not be followed by functional problems, and therefore maybe unrelated to frailty. Given the items do not relate this worsening with functional problems in these areas, this may be an explanation for the poor functioning of these items. Therefore, we removed these items and estimated the CFA again. This time model fit was better and reasonable, as two of the three fit indexes were acceptable: *χ*^2^(51) = 92.38, *p* < 0.001; RMSEA = 0.050; CFI = 0.918; and SRMR = 0.094. Standardized factor loadings for this final model are shown in Fig. 1. The same CFA models were estimated for institutionalized older adults. The one-factor model had a poor fit: *χ*^2^(90) = 143.37, *p* < 0.001; RMSEA = 0.066; CFI = 0.920; and SRMR = 0.135. The three-factor model had a better fit, and two out of three fit indexes were in the acceptable range: *χ*^2^(87) = 119.43, *p* < 0.001; RMSEA = 0.053; CFI = 0.951; and SRMR = 0.124. Nevertheless, items 2, 5, and 13 had very poor factor loadings, and item 13 (live alone) had no variability (was almost constant). Specifically, the standardized factor loadings were: 0.37 (*p* = 0.002) for item 2; 0.39 (*p* < 0.001) for item 5; and 0.21 for item 13 (*p* = 0.11). Substantive reasons for the poor functioning of items 2 and 5 were already mentioned. The case of item 13 in the institutionalized people is clear, they do not live alone by definition, and the item should be avoided in the scale altogether when it is used in this population. Therefore, these items were removed, and a new three-factor model estimated. The new model had a better fit, as only the SRMR was a little above the acceptable cut-off: *χ*^2^(51) = 92.38, *p* < 0.001; RMSEA = 0.050; CFI = 0.910; and SRMR = 0.094. Standardized factor loadings are shown in Fig. 1.

### Reliability estimates

Internal consistencies for the community-dwelling older adults were: alpha for the physical domain = 0.629, 95% CI [0.560, 0.689] with CRI = 0.803; alpha for the psychological domain = 0.410, 95% CI [0.297, 0.508] and CRI = 0.662; and for the social domain, alpha = 0.315, 95% CI [0.174, 0.435] and CRI = 0.518. The estimates for the institutionalized older adults were: for the physical domain, alpha = 0.764, 95% CI [0.696, 0.820] and CRI = 0.894; the psychological domain had an alpha = 0.608, 95% and CI [0.487, 0.705] and CRI = 0.769; and finally, the alpha for the social domain was 0.378, 95% CI [0.126, 0.557] and CRI = 0.682.

### Convergent and divergent validity

Spearman’s correlations were calculated among the three dimensions of the TFI (physical, psychological, and social) and the domains of the alternative frailty scales (Fried’s Frailty Phenotype, Frail Scale, KCL, and Edmonton Scale). As there are several multidimensional instruments and dimensions that somehow relate to the three dimensions in the TFI, convergent validity will show if correlations among clearly related dimensions are large, and divergent validity will show if these correlations are lower for dimensions not so closely related. These correlations are presented in Table 3 (the items with poor behavior were removed prior to calculating the correlations).

**Table 3:** Correlation coefficients of the domains of the Tilburg frailty indicator with alternative frailty measures

	Community-dwelling older adults			Institutionalized older adults		
	TFI physical domain	TFI psychological domain	TFI social domain	TFI physical domain	TFI psychological domain	TFI social domain
Fried’s frailty Phenotype	0.250^†^	0.212^†^	0.038	0.396^†^	0.269^†^	0.200*
FRAIL scale	0.373^†^	0.149^†^	0.060	0.419^†^	0.257^†^	0.149
Edmonton Frailty Scale						
General health status	0.335^†^	0.262^†^	− 0.014	0.452^†^	0.297^†^	0.237^†^
Medication use	0.279^†^	0.172^†^	0.010	0.387^†^	0.333^†^	0.148
Cognition	0.180^†^	0.194^†^	0.126*	0.088	0.165	− 0.090
Functional independence	0.118*	0.039	− 0.109	0.321^†^	0.092	0.150
Social support	0.213^†^	0.206^†^	0.280^†^	0.122	0.146	0.340^†^
Nutrition	0.076	− 0.029	0.007	0.112	0.091	0.154
Mood	0.299^†^	0.533^†^	0.182^†^	0.425^†^	0.622^†^	0.269^†^
Continence	0.221^†^	0.221^†^	0.061	0.172*	0.140	0.164
Functional performance	0.308^†^	0.109	0.090	0.251^†^	0.108	− 0.046
Kihon checklist						
Lifestyle	0.283^†^	0.155^†^	− 0.019	0.097	0.125	0.010
Physical strength	0.484^†^	0.277^†^	0.055	0.377^†^	0.278^†^	0.100
Nutrition	0.023	− 0.089	− 0.064	0.000	0.009	0.088
Eating	0.246^†^	0.146^†^	− 0.012	0.412^†^	0.283^†^	0.112
Socialization /isolation	0.337^†^	0.197^†^	0.049	0.310^†^	0.210*	0.041
Memory	0.238^†^	0.282^†^	− 0.010	0.201*	0.403^†^	0.004
Depressive mood	0.529^†^	0.478^†^	0.110*	0.682^†^	0.476^†^	0.272^†^

TFI Tilburg frailty indicator

*p < 0.05

^†^*p* < 0.001

Convergent validity of the physical domain was fair, it significantly correlated as expected with physical measures, but those correlations were not superior to 0.5. Results related to the analysis of this domain also suggested reasonable divergent validity, showing that the construct of this domain was unrelated with cognitive function (Edmonton cognition, KCL-memory) and social dimensions, except for the KCL-depressive mood and Edmonton mood psychological domains, whose constructs were similar to TFI physical domain in both samples.

The psychological domain correlated with other domains of the alternative frailty instruments, demonstrating some convergent and divergent validity with a similar pattern in both samples. However, the amount of the correlations cannot be considered adequate according to the COSMIN guide. Thus, this domain was related or similar to the psychological domains of the other scales used to compare (Edmonton mood, KCL-depressive mood, KCL-memory) and this psychological dimension was unrelated with physical and social domains.

The social domain suffers from both convergent and divergent validity problems. The correlations of this domain are unrelated with all other measures of frailty. This pattern of correlations does not even meet the COSMIN guideline adequacy criteria for related constructs. Although, in terms of convergent validity, there are related constructs in both samples with Edmonton’s social support dimension, there are unrelated constructs with KCL-socialization/isolation.

## Discussion

Our findings aim to offer further insights on the TFI structure and provide evidence to improve its use in psychometric terms.

The need to confirm the structure of the scale is clear, given the available evidence gaps in some relevant measurement properties [13, 15, 21, 22]. Our results showed that one-factor model is not adequate, with similar results as Vrotsou et al. [13]. The three-factor model showed better fit, but items 2, 5, and 6 showed loadings < 0.40 with the physical domain, as previously showed by other authors [13, 18, 21]. Moreover, previous studies also found low factor loadings for items 13, 15 (social domain) [13, 18], and 14 (social domain) [18, 21]. It must be considered that low factor loadings indicate that the item (indicator) does not relate to the rest of indicators in the factor or dimension, and therefore cannot be aggregated to them. These findings, in line with Vrotsou et al. [13], indicate that the current TFI theoretical structure for the complete scale is not appropriate. Indeed, when certain items were removed, the factor structure was fixed.

Similarly, in the institutionalized sample, the three-factor solution fit better than the one-factor, but only fit well after depurating the poorly behaving items (2, 5, and 13). To our knowledge, no studies have previously analyzed the adequacy of the TFI in institutionalized older adults.

The need to review the TFI model and refine some indicators of the scale has also been suggested by other studies, when analyzing the poor correlations with other items or other similar measures. Among the indicators to be checked, the following have been pointed out: unexplained weight loss (2) [4, 10, 29], poor hearing (5) [10, 29], poor vision (6) [10, 29], ability to cope with problems (12) [10, 11], problems with memory (9) [10], living alone (13) [10], and social support (15) [10]. In the same vein, a recent longitudinal study testing predictive validity of the TFI excluded from the multivariate analyses the indicators poor hearing (5), poor vision (6), feeling down (10), and living alone (13), because they had *p* > 0.20 in the bivariate analyses [30].

A recent systematic review of the psychometric properties of the TFI [22] concluded, despite the 63 validation studies available, the need to continue accumulating evidence on relevant metric properties such as the structural validity to strengthen its use as a clinical decision-making tool. This review included two validation studies, in Spanish [13] and Taiwanese [21]. According to COSMIN guidelines, given the existence of two high-quality studies, the available evidence on the structural validity of this measurement instrument was graded as “sufficient”. However, this concern should be considered with caution. The Spanish validation [13] found poor values in 5 out of 15 items, whereas the Taiwanese validation [21] found very low loadings in 7 items. This was also true for the Turkish validation [18] not included in this systematic review. Therefore, the adequacy of the factor structure of the TFI needs more attention, which is in line with the conclusions of the aforementioned review [22].

Reliability estimates were not equal in both samples, showing better values for the institutionalized older adults. The CRI values for the physical and psychological domains were good (CRI > 0.70) in the institutionalized sample, while only the physical domain was satisfactory in the community-dwelling older adults. In both cases, social domain reliability estimates were not acceptable. These differences between the two groups may be due to a higher mean age, greater variability in the variables or a larger sample of frail people in the institutionalized group. These findings suggest that the TFI seems to be a good assessment tool to detect physical frailty, as indicated by other authors [9]. Furthermore, these findings are in line with the systematic psychometric review of Zamora-Sánchez et al. [22] in which only the TFI physical domain showed sufficient internal consistency of its scores. However, the psychological and social components of this scale should be cautiously considered depending on the context. The indicators of these domains should be carefully analyzed within the construct of frailty. Some items are not homogeneous enough (in terms of covariance) with their intended domains. Perhaps the way the question is written does not highlight the key point, or maybe these indicators could be antecedents or consequences of the process of frailty itself. Therefore, this issue should be studied in detail. Regarding the physical domain, some studies have shown good internal consistency varying from 0.70 to 0.79 [4, 6, 7, 9, 16–18] while others have shown low values varying between 0.57 and 0.68 [10, 11, 13, 14]. Internal consistency was not satisfactory in all studies, with Cronbach’s alpha varying between 0.43 and 0.67 for the psychological and between 0.05 and 0.49 for the social domains [4, 6, 7, 9–11, 13, 14, 16–18]. One plausible explanation could be related to the small number of items of these two domains, as stated by Gobbens et al*.* [4]. However, another possible explanation could be that the components of these domains, especially the social one, do not seem to measure what the scale intends to [18]. The mode of administration of the instrument could also influence the scores’ internal consistency [22].

In addition, although some authors refer to the adequate reliability of the scale considering the estimates of the total TFI [6, 7, 9, 14, 16–18, 21], if we consider that alpha assumes unidimensionality and that the TFI has several dimensions, there is no justification for an overall alpha, but for separate alphas for each dimension.

Convergent and divergent validity for the physical and psychological domains are acceptable given the obtained results, except for some psychological measures whose constructs are similar to the TFI physical domain (KCL-depressive mood and Edmonton mood). These results are in line with several studies in which the construct of alternative psychological measures was related or similar to the TFI physical component [6, 7, 10, 11, 16, 17]. These findings could be explained by the documented relationship between mental health and physical function [7, 17]. Regarding the social domain, our results showed unrelated constructs with most of the alternative measures used. These findings contradict some studies. Nevertheless, when analyzing the values obtained more thoroughly, some of them did not show a clear correlational pattern established in favor of its validity for at least one of the alternative measures related to social dimension, being unrelated (values below 0.30) [6, 10] or related with the rest of the psychological and physical domains [7, 16]. The available evidence shows inconsistent results regarding the association between TFI scores and different variables measuring related or similar constructs [22].

As mentioned before, previous validations have involved community-dwelling older adults. Thus, the use of the TFI in geriatrics still needs to be tested in different settings to explore its potential applications [4, 7, 10, 15, 18, 20, 22]. To the best of our knowledge, this is the first study investigating the validation of the TFI in a sample of institutionalized older adults. These data have been analyzed separately (community-dwelling and nursing homes samples) to compare the results and to assess the validity of the TFI scale as a measure of frailty in institutionalized older adults. Our findings show that a three-factor model is the most suitable one in this context, after removing items in the physical domain (unexplained weight loss and poor hearing) and in the social domain (living alone), since they do not covariate adequately with the other indicators or have no variability. Internal consistency, and convergent and divergent validity were good for the physical and psychological domains. Therefore, the TFI scale could be an acceptable instrument to assess frailty in nursing homes, interpreting the social domain with caution.

## Conclusion

In conclusion, there is a need to revise the TFI structure in more detail and refine some items. Depurating items such as weight loss, poor hearing, and poor vision improve the psychometric characteristics of the scale. The physical domain is a cornerstone as a frailty measure both in community-dwelling and institutionalized older people. However, social component needs further clarification in psychometric terms but also in how it stands within the construct of frailty.

## Data Availability

The datasets generated during and/or analyzed during the current study are available from the corresponding author on reasonable request.
